# Supplementary material for: Loss of the Ap4A hydrolase YqeK impairs stress adaptation and virulence gene expression in Staphylococcus aureus
Source: J Bacteriol. 2026 Aug 20;208(9):e00265-26. doi: 10.1128/jb.00265-26 (PMC13584722; doi:10.1128/jb.00265-26)
Supplement: Supplementary file 1 [file jb.00265-26-s0001.pdf]

Table S1. Strains, plasmids, and primers.

**Strain**	**Description**	**Reference/Source**
S. aureus RN4220	Methicillin-Sensitive Restriction-Deficient *S. aureus*	A. Richardson
S. aureus LAC	Methicillin-Resistant Clinical *S. aureus* Isolate	A. Richardson
MG148	S. aureus LAC Δ*yqeK* (SAUSA300_1552)	This study
MG011	S. aureus LAC + pRMC2	This study
MG162	S. aureus LAC + pRMC2_yqeK	This study
MG166	S. aureus LAC Δ*yqeK* + pRMC2	This study
MG164	S. aureus LAC Δ*yqeK* + pRMC2_yqeK	This study
**Plasmid**	**Description**	**Reference/Source**
pBT2*ts*	*E. coli*/*S. aureus* shuttle vector	(1)
pRMC2	*S. aureus* expression vector	Addgene plasmid #68940 (2)
pBT2_yqeK	5' and 3' homology regions of *yqeK* cloned into the BamHI site of pBT2ts to yield Δ*yqeK*	This study
pRMC2_yqeK	Complete *yqeK* allele with added Shine-Dalgarno cloned into the KpnI/EcoRI sites of pRMC2	This study
**Primer**	**Sequence**	**Use/Source**
p314	CACTA**GGATCC**CCTATTTTAGAGGAAGCAGAGC	*yqeK* deletion (this study)
p315	GCTAATAATTCTTGTGAATTCATTTACATATAATCCTTCCCCCTTAAT	*yqeK* deletion (this study)
p316	ATTAAGGGGGAAGGATTATATGTAAATGAATTCACAAGAATTATTAGC	*yqeK* deletion (this study)
p317	CACTA**GGATCC**CTGCATCTTCATTATGTTCATC	*yqeK* deletion (this study)
p360	CACTA**GGTACC**TACCCGGAGGAGATATTATGAACATTGAAAAAGCAAAACGG	*yqeK* complementation (this study)
p318	CACTA**GAATTC**TTAATCATCCTTTATTCTTTCGTCAC	*yqeK* complementation (this study)

p369	GATACGTAGGTCGTGGTATG	*rpoD* qRT-PCR (3)
p370	CACGAGTGATTGCTTGTC	*rpoD* qRT-PCR (3)
p371	CGATTAGGGATGCAGGTCTT	*agrB* qRT-PCR (this study)
p372	CACCATGTGCATGTCTTCTT	*agrB* qRT-PCR (this study)
p373	CAAGAAGCGATTGACCACAG	*tig* qRT-PCR (this study)
p374	GCTTGTCCACCTTCGAATTC	*tig* qRT-PCR (this study)
p375	GCGAATCTTGCAAGACTTCA	*gatC* qRT-PCR (this study)
p376	AGGTTCAACGCCTTCTGTAT	*gatC* qRT-PCR (this study)
p377	TGGCGTTGTTGGATATGGTA	*spn* qRT-PCR (this study)
p378	TGTCTTTTGTAGCTTGGTCAG	*spn* qRT-PCR (this study)
p379	GCGTCATTAGCGGGTATTTC	*cap8C* qRT-PCR (this study)
p380	TGTGTTATGCGCATCTGAAC	*cap8C* qRT-PCR (this study)
p381	TTTCACCAGACTTCGCTACA	*hla* qRT-PCR (this study)
p382	TCCAGTGCAATTGGTAGTCA	*hla* qRT-PCR (this study)
p383	TGGCACAAGATATCATTTCAACA	*hld* qRT-PCR (this study)
p384	TGAATTTGTTCACTGTGTCGA	*hld* qRT-PCR (this study)
p385	TAGTGGTTTAGCTCAAGGGC	*hrtB* qRT-PCR (this study)
p386	CTCAATTTGCGGCTCTTTCA	*hrtB* qRT-PCR (this study)

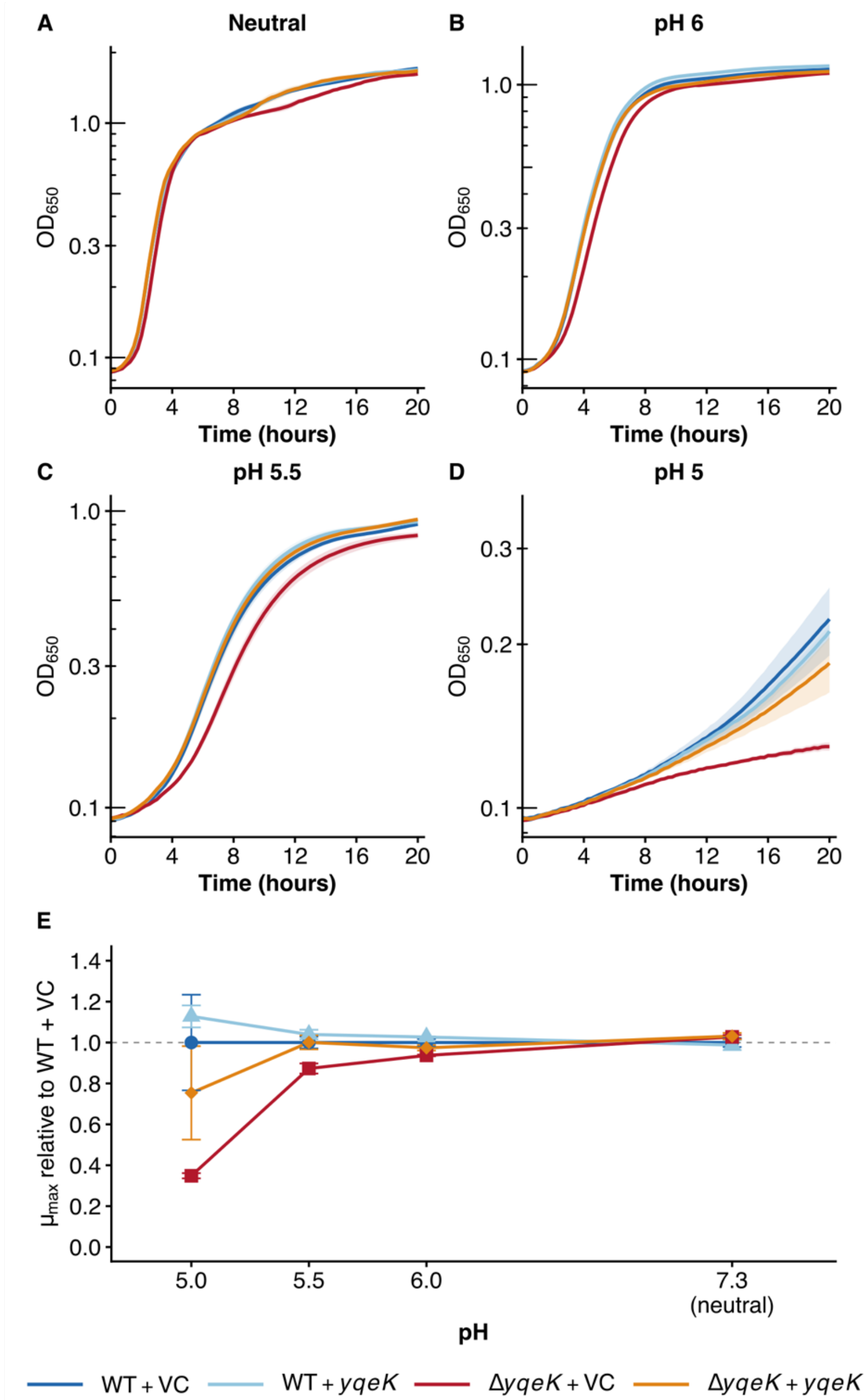

Figure S1. Δ*yqeK* exhibits a larger growth defect relative to WT as pH decreases. Growth curves of the *S. aureus* LAC strains WT + pRMC2 (vector control, VC), Δ*yqeK* + VC, WT + pRMC2-yqeK, and Δ*yqeK* + pRMC2-yqeK (complemented mutant) were performed in 96-well plates either in A) regular TSB (pH ~7.3) or TSB acidified with acetic acid to B) pH 6, C) pH 5.5, or D) pH 5. Growth curves are plotted as means; shaded regions represent SEM. E) Maximum specific growth rates (μ_{max}) were calculated and plotted as a percentage of the μ_{max} of WT + VC under each condition.

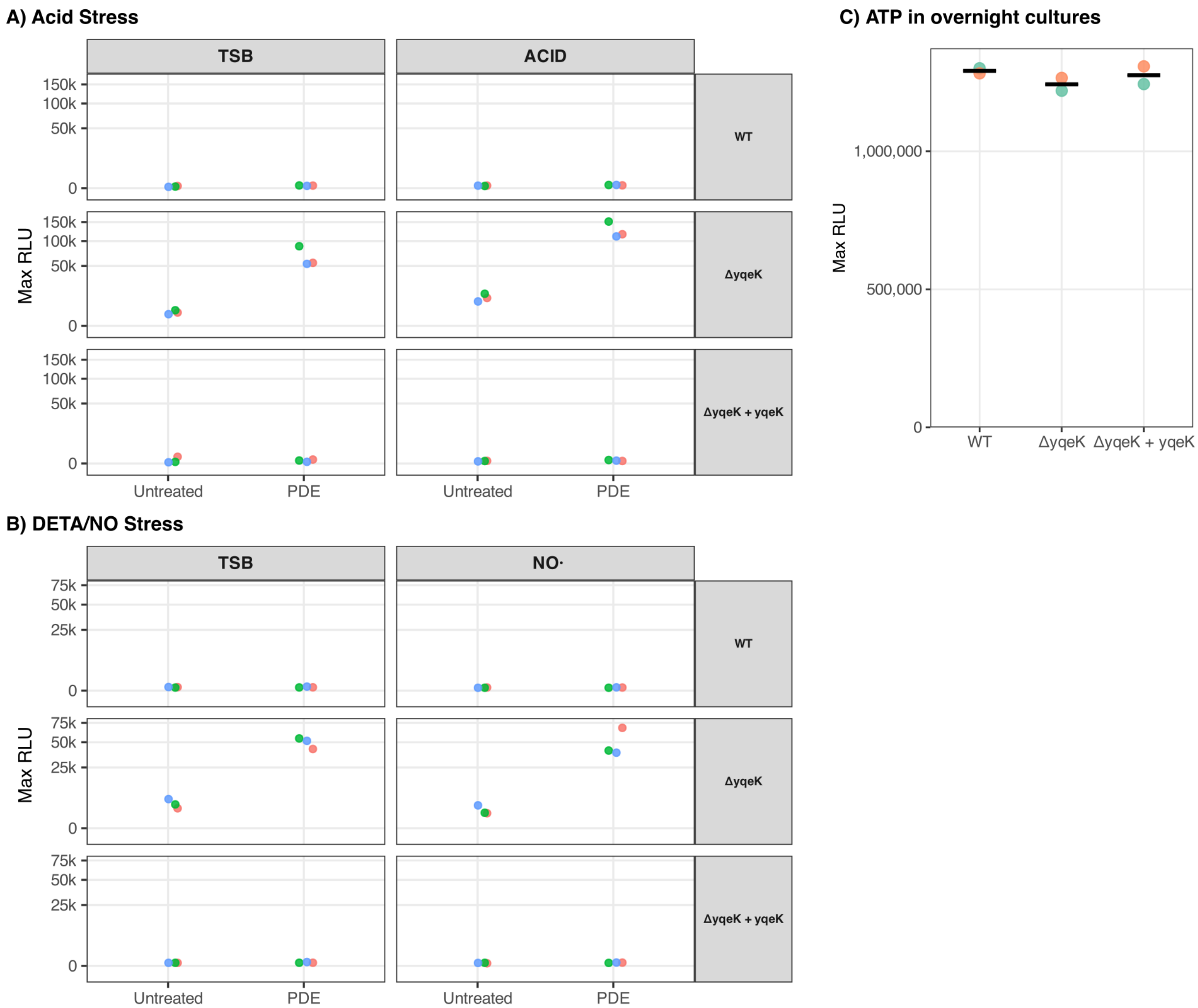

Figure S2. **Supporting data for the Ap_4N luminescence assay.** Raw maximum luminescence (RLU) in ATP-depleted lysates before (Untreated) and after (PDE) snake venom phosphodiesterase treatment are shown for (A) acid stress and (B) NO· stress experiments, prior to OD_{650} normalization and background subtraction used in Figure 3. The near-baseline luminescence in WT and complemented strains regardless of PDE treatment indicates effective ATP depletion across all strain backgrounds, as incomplete depletion would be expected to produce PDE-independent signal elevation in all strains. The elevated luminescence in untreated Δ*yqeK* samples relative to WT is consistent with low-level direct interaction of Ap_4N with the luciferase reagent independent of ATP, as reported in Pojoga et al. 2004 (4). PDE treatment amplified luminescence 12- to 22-fold in Δ*yqeK* lysates compared to 1.1- to 1.2-fold in WT and complemented strains across both stress conditions, confirming that the PDE-dependent signal reflects Ap_4N accumulation rather than residual ATP. Y-axes are on a square root scale. (C) Direct measurement of ATP in overnight cultures prior to phosphatase treatment confirms that elevated pre-PDE Ap4N signal in Δ*yqeK* lysates does not reflect a larger starting ATP pool. BacTiter-Glo reagent was added directly to cultures of each strain at OD_{650} 5.0, without phosphatase or PDE treatment. Each dot represents one biological replicate (n = 2). Horizontal bars indicate means.

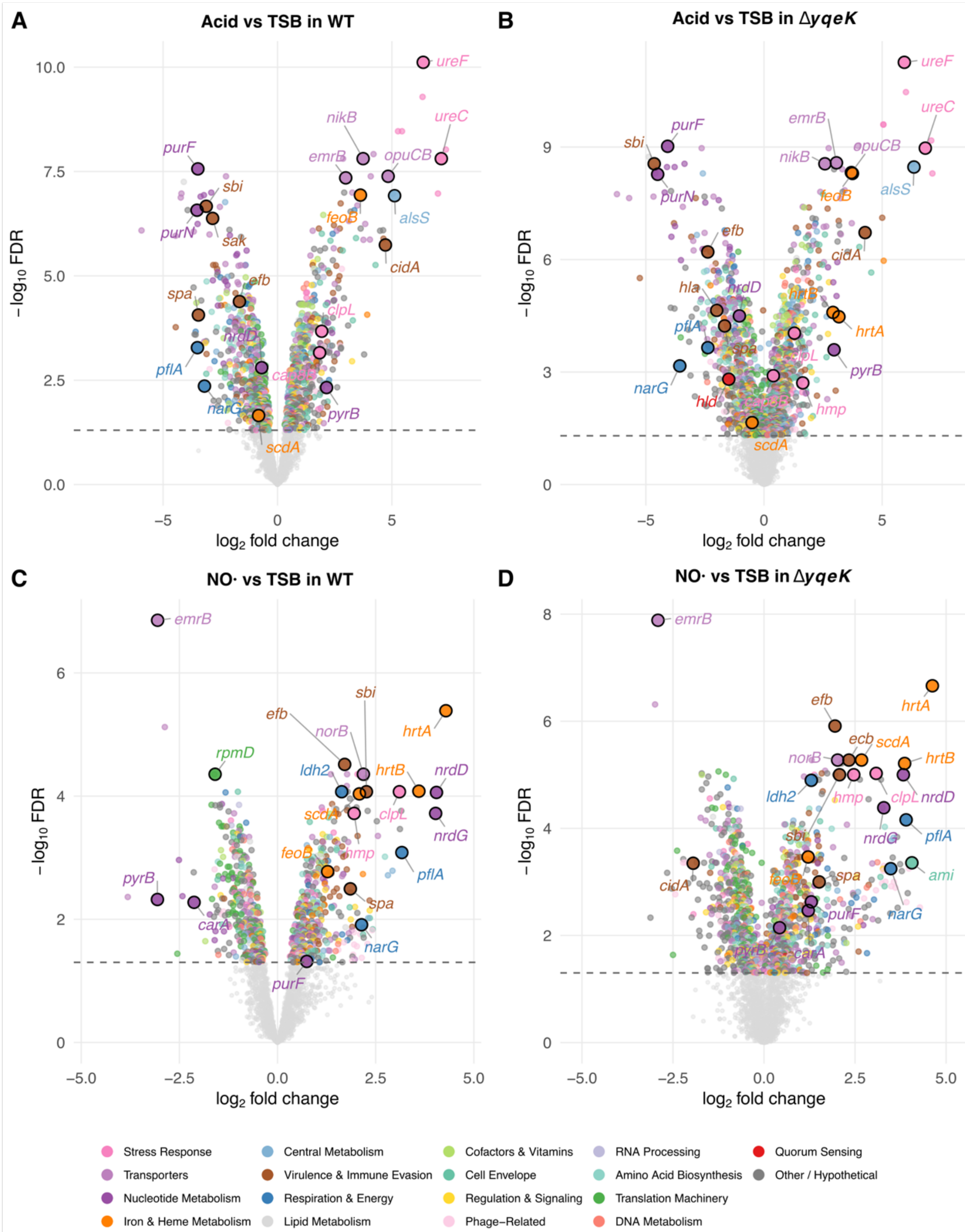

Figure S3. Transcriptome changes within each strain across conditions. Volcano plots showing differential gene expression between growth in acid stress vs. TSB for A) WT and B) Δ*yqeK*, as well as NO· stress vs. TSB for C) WT and D) Δ*yqeK*. Genes were labeled based on a combined significance score ($|\log_2 FC| \times -\log_{10} FDR$) to highlight the most statistically robust differentially expressed genes. Labeled genes were selected from those with annotated functions; hypothetical proteins are plotted but not labeled for clarity.

References

1. Brückner R. 1997. Gene replacement in Staphylococcus carnosus and Staphylococcus xylosus. FEMS Microbiol Lett 151:1–8.

2. Corrigan RM, Foster TJ. 2009. An improved tetracycline-inducible expression vector for Staphylococcus aureus. Plasmid 61:126–129.

3. Theis T, Skurray RA, Brown MH. 2007. Identification of suitable internal controls to study expression of a Staphylococcus aureus multidrug resistance system by quantitative real-time PCR. J Microbiol Methods 70:355–362.

4. Pojoga LH, Moose JE, Hilderman RH. 2004. Characterization of the interaction of P1,P4-diadenosine 5′-tetraphosphate with luciferase. Biochem Biophys Res Commun 315:756–762.
